# Religious Coping and Life Satisfaction during the COVID-19 Pandemic among Polish Catholics. The Mediating Effect of Coronavirus Anxiety

**DOI:** 10.3390/jcm10214865

**Published:** 2021-10-22

**Authors:** Paweł Piotr Dobrakowski, Sebastian Skalski, Janusz Surzykiewicz, Jolanta Muszyńska, Karol Konaszewski

**Affiliations:** 1 Institute of Psychology, Humanitas University, 41200 Sosnowiec, Poland; 2 Institute of Psychology, Polish Academy of Sciences, 00950 Warsaw, Poland; sebastian.skalski@sd.psych.pan.pl; 3 Faculty of Philosophy and Education, Catholic University of Eichstaett-Ingolstadt, 85051 Eichstaett, Germany; janusz.surzykiewicz@ku.de; 4 Faculty of Education, The Cardinal Wyszynski University in Warsaw, 01815 Warsaw, Poland; 5 Faculty of Education, University of Bialystok, 15328 Bialystok, Poland; jolamusz@uwb.edu.pl (J.M.); k.konaszewski@uwb.edu.pl (K.K.)

**Keywords:** religious coping, life satisfaction, COVID-19, fear of COVID-19

## Abstract

Recent data have indicated that people may have experienced fear during the COVID-19 pandemic. This study aims to deepen our understanding of the relationship between religious coping and life satisfaction by analysing the indirect effects of fear of COVID-19. Methods: This study included 365 people (75% women) aged 18–78 years. The procedure consisted of completing questionnaires to measure religious coping, COVID-19 anxiety, satisfaction with life, and satisfaction with social support. Results: Structural equation modelling showed that positive religious coping was related to greater life satisfaction and greater satisfaction with social support during the pandemic. Moreover, fear of COVID-19 mediated the relationship between negative religious coping and life satisfaction and social support satisfaction. Conclusions: The data suggest a need for practitioners to focus on interventions that enhance positive religious coping to improve life satisfaction during the spread of infectious diseases.

## 1. Introduction

The COVID-19 pandemic has resulted in serious changes to the geopolitical/economic context and all areas of social life. It has significantly reduced life satisfaction and well-being, and has affected the mental health of individuals and their immediate environment [1]. As a result, there is increasing research concerning mental health during the pandemic. Much of this research is focused on disturbances to psycho-emotional and social functioning, or increased susceptibility to mental health problems and suicidal behaviours. Numerous stressors have been identified that can lead to severe anxiety [2,3,4,5,6]. Recent data indicate that people in quarantine experienced anxiety, anger, confusion, and stress [7,8,9,10]. Relatively high rates of anxiety (6.33–50.9%), depression (14.6–48.3%), post-traumatic stress disorder (7–53.8%), psychological distress (34.43–38%), and stress (8.1–81.9%) have been reported in the general population during the COVID-19 pandemic in China, Spain, Italy, Iran, the United States, Turkey, Nepal, and Denmark [11]. This has led researchers from various fields of science to initiate an empirical exploration of these issues, ranging from typical medical rehabilitation and care research concerning the phenomenon to studies related to personal life, satisfaction, quality of life, and the social environment. The latter scope is essential for psychosocial support and education [12,13,14]. From the perspective of developing proper support concepts, it is important to correctly identify and activate the available psychosocial capabilities (resources) and means to assist in designing supportive interactions [15,16,17].

Many studies have demonstrated the relationships between the broadly understood psychosocial functioning of individuals, their health, well-being, and quality of life. However, little is known about the combined effect of religious coping and perceived fear of COVID-19 on life satisfaction. To date, the mediating effect of COVID-19 anxiety on the relationship between religious coping and life satisfaction has not been taken into consideration. Moreover, so far little research has focused on the level of life satisfaction of adult Christians Poles experiencing the COVID-19 pandemic, as well as factors (e.g., fear of the coronavirus and religious coping) that could be related to life satisfaction during that time. Christianity is the dominant religion in Poland, and about 90% of the population considers themselves as Christian [18]. The results of such research could contribute to the development of interventions and therapeutic programs (e.g., resource and meaning-based approaches that address existential anxiety and mental well-being as well as individual and social life satisfaction). It is difficult to find people and cultures where maintaining health and life satisfaction is not important in difficult times such as during the COVID-19 pandemic. From the perspective of developing proper support concepts, it is important to properly identify and activate the available psychosocial capabilities and resources to assist in designing supportive interactions [15,17].

### 1.1. Life Satisfaction and Religious Coping

The growing number of COVID-19 cases and deaths, fear of job loss, and significant restrictions in terms of public life, in addition to the economic downturn, have negatively affected life satisfaction [19,20]. Life satisfaction is understood as a measure of how positively a person assesses the overall quality of their life as a whole [21,22]. It refers to the subjective assessment of the current quality of life, which is an important indicator of mental health and well-being [21]. It constitutes a key personal force for promoting well-being and increasing vitality and preventing psychopathology as well as susceptibility to disease [23,24,25]. From the perspective of social policy, it is important to identify determinants of life satisfaction during pandemics. Research has shown that life satisfaction has served as a protective factor against psychological distress during the COVID-19 pandemic [15,26,27]. In terms of exploring the determinants of life satisfaction, many researchers point to the role of religion and spirituality [20].

During the past three decades, numerous scientific studies have confirmed the special role of religion and spirituality in enhancing life satisfaction [20,28,29,30]. In terms of COVID-19, the relationships between spirituality/religious practices and strategies related to COVID-19 can be confirmed with regard to anxiety, depression, and positive mental health outcomes including life satisfaction and well-being [31,32,33,34,35,36,37,38]. This perspective of perceiving the issues directs researchers to study the reasons for this maintenance or improvement of mental health and life satisfaction. Research highlights, among other aspects, that religion and spirituality are necessary sources that activate individual coping strategies in order to support individuals in developing life satisfaction [39,40]. Religiousness and the ability for religious coping can be considered as protective factors when individuals struggling with their fears and concerns have an absolute trust in God and when they express patience and gratitude under all circumstances, including those of sorrow and worry [32,41,42]. Furthermore, developing deeper religious faith through prayer, reading holy books, and listening to inspirational programs may help individuals stay mentally healthy, as it has been reported that religious practices are associated with less anxiety and stress, as well as with greater hope [28]. Accordingly, religious coping as part of a broader coping construct may be working in favour of life satisfaction during the COVID-19 pandemic.

An important predictor of life satisfaction consists of coping, which is considered a key factor in psychosocial adaptation [43,44,45]. It is a significant element that is important in the context of an individual’s personality traits and consists of the basic elements of human cognitive, emotional, and social functioning [46]. Therefore, dealing with everyday challenges and long-term developmental outcomes plays a key role in how the individual deals with them on a daily basis. The repertoire of coping strategies also includes activities that relate to the spiritual-religious sphere (religious positive coping and religious negative coping). Religious forms of coping with stress constitute specific ways of confronting difficult situations through a reference to God and faith, not only in the religious context, but also from the perspective of activity in the spiritual sphere of individuals [47,48,49,50,51]. Usually, only some strategies included in an individual’s repertoire are involved in the coping process. Their selection is determined by the situation that the individual is dealing with as well as by the personality traits possessed by that individual. The general opinion states that coping is important in terms of achieving or maintaining good health, well-being, and life satisfaction. With regard to this concept, researchers also point out that religious coping can predict results that are important in terms of promoting aspects of health, quality, and life satisfaction [29,52,53]. Taking into consideration the empirical data and theoretical framework, religious coping seems to be crucial in terms of promoting mental health and life satisfaction. Importantly, this applies to followers of various religions, including Christians [54,55], Muslims [56,57], Buddhists [58] and Hindus [59], as well as people identifying as non-religious [60] and not spiritual [61,62]. Despite the obvious significance of religion for individuals and society, psychologists and other social scientists have paid relatively little attention to religious coping in the empirical literature [52].

In previous studies, negative religious coping was associated with lower overall levels of well-being, life satisfaction, and quality of life [53,63,64,65]. Most studies have shown that positive religious coping promotes high levels of life satisfaction and well-being [53,65,66]. Only in a few cases has the association between the two variables been insignificant [63,64]. Furthermore, negative religious coping was associated with lower levels of quality of life in specific domains, including with poorer physical functioning, vitality, social functioning, and mental health [67]. Moreover, in terms of studies carried out in clinical groups (including cancer patients) undergoing spiritual-focused therapy, it was found that taking advantage of positive religious methods of coping with stress is accompanied by a higher level of physical well-being and a lower severity of depressive symptoms and anxiety, while more frequent use of negative religious ways of coping with stress is associated with a deficit in mental well-being, as well as increased depression and anxiety [68]. So far in the literature, it has been found that positive religious coping constitutes a protective factor for a number of mental disorders, including depression, anxiety, and PTSD [69,70]. It has also been found that positive religious coping increases the quality and satisfaction with life [67,71,72]. Positive forms of religious coping also show positive relations with a higher level of life satisfaction, for example among patients with chronic diseases [73,74,75], while negative religious coping results in a lower level of life satisfaction [29,67,76]. Significant associations between positive and negative religious coping and life satisfaction have also been shown in other reports [44,77,78].

### 1.2. The Anxiety of COVID-19 as a Mediator between Religious Coping and Life Satisfaction

The literature concerning the relation between religious coping during the COVID-19 pandemic and life satisfaction as an indicator of mental health has focused mainly on the direct relationship between these variables. As anxiety may constitute a significant phenomenon affecting life satisfaction under these conditions, it seems important to examine the mediating role of this variable in terms of the relationship between religious coping and life satisfaction. Over the past 20 years, over 100 empirical studies have analysed the relationship between religious struggles as well as religious coping and symptoms of anxiety [79]. Studies in this area are generally consistent and show that the positive aspects of religious coping and religious struggles protect against the occurrence and severity of such symptoms (to varying degrees), while negative aspects of religious coping and religious struggles constitute risk factors for general mental health [79].

Studies so far have shown that the COVID-19 epidemic is a source of anxiety for people around the world [80,81,82]. The abovementioned reports treat anxiety in terms of a physiological process. The following construct describes the physiological response induced by a sense of threat and a nonspecific feeling of mental tension, accompanied by the activation of the autonomic nervous system and a number of psychosomatic symptoms [83]. It is believed that fear of the spread of an infectious disease constitutes an indicator of mental function during the pandemic [84,85,86]. It is associated with depression and post-traumatic stress disorder, as well as the post-traumatic growth, life satisfaction, and well-being of an individual [26,34,87,88]. Therefore, it seems necessary to identify the psychosocial resources that may be associated with an increasing fear of COVID-19. Religious coping may prove to be one such resource [89]. Previous reports have shown that negative religious coping increases the severity of anxiety, while positive coping has the opposite effect, i.e., it reduces anxiety [52,90]. However, it should be noted that not all reports have shown a significant relationship between positive religious coping and anxiety [91]. Further studies are needed to better understand these dependencies.

The association between negative religious coping and greater levels of anxiety has also been seen in COVID-19 research [32,80,87]. According to metacognitive models, negative beliefs concerning uncertainty cause difficulties in coping with situations that cause uncertainty, which may lead to excessive worrying and anxiety [92]. Furthermore, researchers observed that fear of COVID-19 mediated the effects of personality (resilience) and environmental (social support) variables on psychosocial functioning during the pandemic, as well as on the effects of trauma [81,85,93]. The conceptual model of *coronaphobia* assumes that the outbreak of the pandemic has forced a change in coping methods and mechanisms [94]. Such an attack on temporal stability can lead to an outburst of irrational and negative emotions, i.e., panic, anxiety, and phobias [95]. It is assumed that factors shaping the fear of the coronavirus disrupt everyday life and reduce life satisfaction [94]. Due to this, it seems that the fear of COVID-19 may mediate the relation between religious coping and life satisfaction. Similar studies have not yet been conducted.

### 1.3. Study Purpose

This study aims to deepen our understanding of the relation between religious coping and life satisfaction by analysing the indirect effects of the fear of COVID-19. Based on the literature described above, we assume that: (a) overall life satisfaction will be related with positive religious coping and will be negatively associated with negative religious coping; (b) satisfaction with social support will be related to positive religious coping and will be significantly related to religious negative coping; (c) positive religious coping will be associated with a lower level of COVID-19 anxiety and negative religious coping will be associated with a higher level of anxiety; (d) fear of COVID-19 will be associated with lower levels of life satisfaction and satisfaction with social support; and (e) fear of COVID-19 may mediate in the relation between positive and negative religious coping and life satisfaction and satisfaction with social support.

## 2. Method

### 2.1. Subjects and Procedure

The study was carried out from April to September 2020 with the consent of the Ethics Committee of the Institute of Psychology, Polish Academy of Science (# 31/III/2020). Sample selection was purposeful. The study included adults who defined themselves as practicing Catholics. During the recruitment process candidates declared their participation in Catholic rites. No additional recruitment criteria were required. This study included 365 people (75% women and 25% men) aged 18–78 years (*M* = 35.64; *SD* = 14.55). It should be noted that the obtained sample is not representative of the Polish population, where we would expect 52% women and an average age of 43 years. The invitation to participate in the study was sent using social media and websites. The Google Forms platform was used to collect data. Each participant provided conscious consent to participate in the study anonymously. The procedure consisted of questionnaire completion to measure religious coping, COVID-19 anxiety, satisfaction with life, and satisfaction with social support.

### 2.2. Measures

The RCOPE Brief was used to measure religious coping. This is a 14-item questionnaire assessing the degree to which a given person takes advantage of certain methods of religious coping [96]. It consists of 2 factors: (1) positive religious coping, which includes seeking spiritual support, seeking spiritual connection, cooperating with God in terms of solving problems, religious forgiveness, and a sympathetic religious appreciation of illness; and (2) negative religious coping, consisting of perceiving God in terms of punishments, interpersonal religious discontent, demonic judgments, spiritual discontent, and questioning God’s power. People indicate how often they engage in any form of religious coping on a 4-point scale from 0 (not at all) to 3 (a lot). This tool shows good validity and a satisfactory internal consistency (for positive coping α = 0.86, and for negative α = 0.74 for data in the Polish language version [97]). In the study, the reliability of the scale for positive religious coping was α = 0.94, while for negative religious coping this value was α = 0.85.

The Coronavirus Anxiety Scale (CAS) constitutes a 1-way tool designed to assess the severity of anxiety related to the mental crisis resulting from the coronavirus pandemic [80]. The participant responds to 5 statements (symptoms of anxiety) on a 5-point Likert scale, where 0 = “Not at all” and 4 = “Almost every day”. Research carried out among adults living in the United States has shown that CAS is a diagnostically accurate and reliable tool for assessing the severity of coronavirus anxiety. CAS results were statistically significantly correlated with general anxiety, depression, and suicidal thinking, as well as drug and alcohol use. The diagnostic properties of the scale (sensitivity: 90%, specificity: 85%) proved to be comparable to those of other screening tools such as the Generalised Anxiety Disorder-7. The Polish language version showed a satisfactory accuracy and internal consistency (*α* = 0.86) [81]. In the conducted study, the reliability of the scale was at *α* = 0.90.

The Brief Multidimensional Life Satisfaction Scale (BMLSS) consists of 8 statements for measuring the overall satisfaction with life and 4 statements for measuring satisfaction with social support. General satisfaction with life consists of various domains, for example the internal (me, all of life), social (friendships, family life), external (work, where I live), professional (financial situation, future prospects), and health-related (health situation) domains, as well as the ability to deal with everyday life. Satisfaction with social support concerns the sense of support received from friends, relatives, and acquaintances. The participant expresses his or her attitude towards the statements on a 7-point scale, where 1 = “very dissatisfied” and 7 = “very satisfied”. Scores > 50% indicate high life satisfaction, while scores < 50% indicate low satisfaction. The 8-item scale (BMLSS) has good reliability (α = 0.87) [98]. In terms of the conducted study, the reliability of the life satisfaction scale (BMLSS) was at α = 0.91, while the reliability of the social support satisfaction scale (BMLSS Support) was α = 0.87.

### 2.3. Statistical Analysis

The r-Pearson correlation coefficient was used to determine the relationship between the variables, which allows us to know the strength and shape of the linear relationship between 2 variables. Values between 0 and 0.30 were interpreted as representing small correlation, 0.3 to 0.5 as a moderate correlation, 0.50 to 0.70 as a large correlation, and values between 0.70 and 1 as a very large correlation [99]. Next, we used structural equation modelling (SEM) to search for relations between the variables. The analysis of structural equations was carried out with the use of the AMOS program. Model parameters were estimated with the use of the maximum likelihood method. To assess the model’s proper adjustment to the data, the following indices were used: goodness-of-fit index (*GFI*), comparative fit index *(CFI*), root-mean-square error of approximation (*RMSEA*), and relative chi-squared (*χ*^2^/*df*). *GFI* values ≥ 0.90 and *CFI* values ≥ 0.95 indicate good and adequate adjustment of the model to the data [100]. *χ*^2^/*df* values < 2 also suggest a good fit of the model to the data. *RMSEA* values < 0.08 can also be interpreted as a good fit to the data [101,102,103]. To verify the mediating role of COVID-19 anxiety on the relationship between religious coping and life satisfaction/social support satisfaction, a bootstrapping analysis (for 2000 samples) was carried out to establish 95% percentile confidence intervals for the estimated effects. When the value of the confidence intervals exceeds 0, it means that the given effect is insignificant.

## 3. Results

Table 1 presents the descriptive statistics. The mean results obtained in the study group in terms of life satisfaction indicate moderately high satisfaction with life (*M* = 39.22; *SD* = 12.48) and relatively low satisfaction with social support (*M* = 11.17; *SD* = 6.27). The average level of COVID-19 anxiety was low (*M* = 1.65: *SD* = 3.36) which fits within the limits of the fourth sten and indicates a moderate level of this phenomenon among the participants of the study. The mean score on the scale of positive religious coping (*M* = 15.68; *SD* = 7.48) fits within the limits of sten 5 and can be defined as average coping, while the scale of negative religious coping (*M* = 9.77; *SD* = 4.11) is within the third sten, describing as a low tendency towards taking advantage of this means of coping.

The analysis of the correlations showed significant and positive interrelations between satisfaction with life and satisfaction with social support (*r* = 0.62. *p* < 0.001). The association between life satisfaction and COVID-19 anxiety was negative (*r* = −0.21. *p* < 0.001). Satisfaction with social support was also significantly and negatively related with COVID-19 anxiety (*r* = −0.14, *p* < 0.001). There was a positive correlation between life satisfaction and positive religious coping (*r* = 0.15, *p* < 0.001), and a negative correlation between negative religious coping and life satisfaction (*r* = −0.24, *p* < 0.001). The relationship between satisfaction with social support and negative coping was insignificant (*r* = −0.06, *p* > 0.050) and positive for positive religious coping (r = 0.33, *p* < 0.001). Relationships between religious coping strategies were also noted—the relationship between positive and negative strategies (*r* = 0.23, *p* < 0.001) was found to be positive. COVID-19 anxiety was positively correlated with positive (*r* = 0.13, *p* < 0.01) and negative religious coping (*r* = 0.22. *p* < 0.001) (see Table 1).

**Figure 1:**
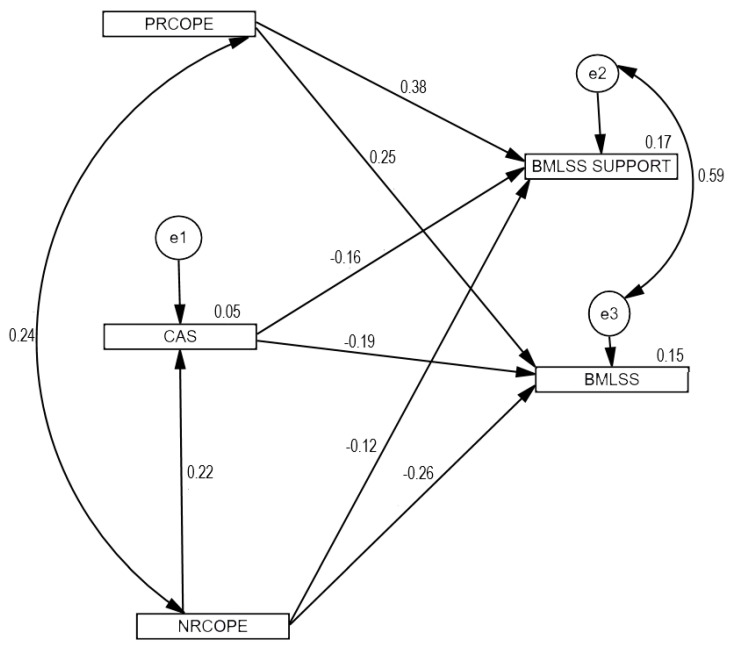
Model tested: religious coping and COVID-19 anxiety, as well as satisfaction with life and satisfaction with social support.

Then, structural equation modelling was used to verify the basic hypotheses. The relationships between positive and negative religious coping, COVID-19 anxiety, and life satisfaction as well as satisfaction and social support were analysed. The first tested model included eight paths representing the eight hypotheses. The model fit was unacceptable: *χ*^2^(2) = 176.26; *p* < 0.001; *χ*^2^/*df* = 88.13; *RMSEA* = 0.489 (low = 0.430; high = 0.552; 90% CI); *GFI* = 0.86; *AGFI* = 0.04; *CFI* = 0.43. Due to the previously established relationships between the analysed variables, apart from the regression paths necessary to examine the predictive value of the variables, in the second tested model we included the covariance between positive and negative religious coping as well as the correlation between life satisfaction and satisfaction with social support. The tested model contained seven regression paths included in the determined hypotheses. The relation between positive religious coping and COVID-19 anxiety was insignificant, so this path was removed from the model. The model was found to be well suited to the data: *χ*^2^(1) = 2.66; *p* = 0.103; *χ*^2^/*df* = 2.66; *RMSEA* = 0.068 (low = 0.000; high = 0.172; 90% CI); *GFI* = 0.997; *CFI* = 0.995. Figure 1 presents the standardized path coefficients: for one-direction arrows these are standardised regression coefficients, and for two-direction arrows these are correlation coefficients. Combined positive and negative religious coping as well as COVID-19 anxiety explained 15% of the variance concerning life satisfaction and 17% of the variance concerning satisfaction with social support.

There were positive direct effects of positive religious coping on life satisfaction (*β* = 0.25, *p* < 0.001) and satisfaction with social support (*β* = 0.38, *p* < 0.001). The direct effects of negative religious coping on life satisfaction were negative (*β* = −0.26, *p* < 0.001), and this was also the case for the sense of satisfaction with social support (*β* = −0.12, *p* < 0.050). A direct relationship between religious negative coping and COVID-19 anxiety was also confirmed, and the effect was positive (*β* = 0.22, *p* < 0.001). Table 2 presents the results of the coefficients of the final model. Furthermore, the results of the mediation analyses indicate that fear of COVID-19 plays a mediating role both in the relationship with life satisfaction and with satisfaction with social support. There was a partial mediation effect here. Table 3 presents the results of the specific indirect effects included in the model.

For comparison, we examined a model in which anxiety could mediate the relationship between positive religious coping and life satisfaction. The model fit was unacceptable: *χ*^2^(2) = 14.809; *p* < 0.001; *χ*^2^/*df* = 14.809; *RMSEA* = 0.195 (low = 0.115; high = 0.288; 90% CI); *GFI* = 0.98; *AGFI* = 0.76; *CFI* = 0.95.

## 4. Discussion

The aim of this study was to assess the relationships between religious coping, fear of COVID-19, life satisfaction, and satisfaction with social support. As expected, positive religious coping was related to greater life satisfaction and greater satisfaction with social support among Polish Catholics during the COVID-19 pandemic. Our observations highlight that certain aspects of religion can have a key impact on adaptation during times of crisis, as suggested by previous research [28,33,36,40,71,104,105]. It seems that religiousness encompasses a framework for assigning meaning which is related to reduced mental distress and the pursuit of mental well-being [28,106,107]. It should be emphasized that in our study we did not assess the level of intensity of religious identity. According to Aten and colleagues, this variable may increase the relationship between positive religious coping and well-being, because any turn towards faith implies a stronger sense of belonging to a group [104]. Our study confirmed the relationship between positive religious coping and satisfaction with life and with social support. Our research noted not only positive weak effects of positive religious coping on life satisfaction, but also moderate positive effects of positive religious coping on satisfaction with social support. Therefore, it can be concluded that shaping and taking advantage of this coping strategy has a significant impact not only on general life satisfaction, but also on satisfaction with social support [77,108,109].

Negative religious coping was related to lower life satisfaction and satisfaction with social support. Our findings correspond with reports stating that that negative religious coping is a predictor of poor mental adjustment, mental health, and low life satisfaction [63,110]. Negative religious coping was also positively related to the level of COVID-19 anxiety. In an earlier study by Pirutinsky and colleagues, negative religious coping was related with fear of exposure to COVID-19, increased stress, and lower levels of positive impact [36]. Therefore, it seems that negative religious coping may be dysfunctional in terms of mental health during a pandemic.

The fear of COVID-19 mediated the relations between negative religious coping and life satisfaction and social support satisfaction. In other words, people taking advantage of negative religious coping were more likely to experience fear of COVID-19, which in turn was related to a lower level of life satisfaction and social support. The obtained effects correspond with data in the literature to date, according to which the fear of the spread of an infectious disease constitutes a marker of psychosocial functioning during a pandemic and mediates the impact of psychological variables on the effects of trauma [81,85,88].

The obtained data unexpectedly showed that positive religious coping was correlated with a higher severity of COVID-19 anxiety. Our finding is inconsistent with the consensus found in the literature stating that positive religious coping is usually related to reduced symptoms of anxiety [52,90]. To fairly explain this dependence, it should be noted that some priests in Poland denied the existence of the pandemic, which could have caused existential dissonance among the faithful. In such a situation, anxiety constitutes the result of emerging discrepancies between the acquired knowledge and the incorrect and redundant information coming from the environment [111]. Moreover, fear may have increased when God did not listen to prayers for an end to the pandemic. Furthermore, strong anxiety could have resulted in increased positive religious coping to deal with the stressor.

Most of the obtained effects were found to be weak or moderate; however, in our opinion, they are important. In previous studies, positive interventions showed greater efficacy than other techniques designed to improve well-being [112]. It should be added that this means strengthening the potential of healthy people during the spread of an infectious disease, and not alleviating symptoms or disorders among infected people or health care workers. Moreover, what is measured as satisfaction with life may differ from what individuals generally consider to be genuine happiness or lasting personal fulfilment, and from what may constitute elements of personal well-being [113,114].

The presented positive impact of positive religious coping on the sense of satisfaction with life in both dimensions indicates the need to shape and develop them. Building a construct may contribute towards improving the life satisfaction of adults. For example, gaining new experiences may work in favour of developing them. Positive religious coping can also be supported by stimulating the motivation to undertake new actions or change behaviour, e.g., by fostering self-esteem, efficiency, independence, flexibility, creativity, and spirituality. Thus, it is worth including psychoeducation aimed at strengthening resources and competences through interventions aimed at adults [115,116,117]. Diagnosing and understanding the mechanisms related to religion in the field of adult education, as a quality that is both performative as well as variable, raises all kinds of questions and challenges for teaching. Teaching practice thus becomes a part of a shared growth and challenge, and anxiety should be recognised and boldly discussed without trying to depreciate it or protect adults from it. It seems necessary to develop various identities in the learning process and provide ways of acquiring skills leading to the application of many techniques in various fields, both in a literal and philosophical sense. The above may allow for proper psychosocial functioning and life satisfaction.

In this study, positive religious coping improved life satisfaction during COVID-19. On the other hand, it should be noted that religious convictions (in addition to lack of knowledge, lack of awareness, past experiences, the perceived importance of vaccinations, subjective norms, residence in rural areas, reduced trust in government and pharmaceutical industry, and the passing of time in a pandemic) increase vaccine hesitancy [118,119]. The findings from the cited studies can be used to formulate health policies related to COVID-19 vaccination. In Poland, for example, Catholic and Orthodox clergy have joined together to promote vaccination, and parish vaccination centres have been established near places of worship.

### Limitations

The cross-sectional nature of the study does not allow for unequivocal statements concerning causes and effects. Because the variables were not measured at two time points (before and during the pandemic), the effect of the pandemic outbreak on the relationship between religious coping and life satisfaction cannot be clearly determined. The sample was obtained through a snowball procedure, which makes broader generalizations difficult. Only Polish Catholics participated in the study, which makes it impossible to relate the data to other religious groups. Furthermore, the participants were neither convalescents nor in the active phase of a COVID-19 infection; results in these groups may differ from those of the general population. In future research it would be interesting to take advantage of experimental techniques, e.g., manipulation in the field of applied religious coping strategies. The work would have been more interesting if other groups from other countries with different spiritual positions or levels of spirituality had been analysed. In addition, in future research it would be pertinent to consider the measurement of other variables that would account for greater variance in terms of life satisfaction.

## 5. Conclusions

The present study is one of the first to assess the associations between religious coping, COVID-19 anxiety, life satisfaction, and satisfaction with social support. Findings suggest that future psychological interventions should aim to develop positive religious coping, which may promote life satisfaction and satisfaction with social support during the spread of infectious diseases. The data also indicate that negative religious coping and fear of COVID-19 may be dysfunctional for mental health during a pandemic.

## Figures and Tables

**Table 1:** The matrix of correlation for negative and positive religious coping and fear of COVID-19 with life satisfaction and social support satisfaction.

	1.	2.	3.	4.	5.
1. BMLSS	-				
2. BMLSS SUPPORT	0.62 **	-			
3. CAS	−0.21 **	−0.14 **	-		
4. PRCOPE	0.15 **	0.33 **	0.13 *	-	
5. NRCOPE	−0.24 **	−0.06	0.22 **	0.23 **	-
*M*	39.22	11.17	1.65	15.68	9.77
*SD*	12.48	6.27	3.60	7.48	4.11

* *p* < 0.050, ** *p* < 0.010.

**Table 2:** The results of the coefficients of the final model.

	*β*	B	SE	C.R.	*p*
NRCOPE > CAS	0.22	0.165	0.038	4.390	***
CAS > BMLSS	−0.19	−0.725	0.186	−3.895	***
RRCOPE > BMLSS SUPPORT	0.38	0.352	0.045	7.746	***
PRCOPE > BMLSS	0.25	0.430	0.087	4.942	***
NRCOPE > BMLSS	−0.26	−0.712	0.141	−5.060	***
CAS > BMLSS SUPPORT	−0.16	−0.327	0.097	−3.358	***
NRCOPE > BMLSS SUPPORT	−0.12	−0.171	0.073	−2.325	0.020

Annotations—*β*: standardized regression coefficient; B: non-standardized regression coefficient; SE: standard errors; C.R.: critical ratios and *p*-values (*** *p* < 0.001).

**Table 3:** Indirect effects of negative religious coping and fear of COVID-19 on life satisfaction and satisfaction with social support.

			95% CI	
	*β*	B	LL	UL
NRCOPE > CAS > BMLSS_SUPPORT	−0.04	−0.05	−0.10	−0.03
NRCOPE > CAS> BMLSS	−0.04	−0.12	−0.22	−0.06

Annotations—*β*: standardised regression coefficient; B: non-standardised regression coefficient; BootLL and BootUL: lower and upper confidence limits, respectively, for the bootstrapping method.

## Data Availability

The data presented in this study are available on request from the corresponding author.
